# Role of Adjuvant Chemotherapy in Advanced Stage Upper Urinary Tract Urothelial Carcinoma after Radical Nephroureterectomy: Competing Risk Analysis after Propensity Score-Matching

**DOI:** 10.7150/jca.34103

**Published:** 2019-11-17

**Authors:** Minyong Kang, Heejin Yoo, Kyunga Kim, Si Hyun Sung, Hwang Gyun Jeon, Se Hoon Park, Seong Il Seo, Seong Soo Jeon, Hyun Moo Lee, Han Yong Choi, Byong Chang Jeong

**Affiliations:** 1 Department of Urology, Samsung Medical Center, Sungkyunkwan University School of Medicine;; 2 Department of Health Sciences and Technology, SAIHST, Sungkyunkwan University;; 3 Statistics and Data Center, Samsung Medical Center;; 4 Division of Hematology-Oncology, Department of Internal Medicine, Samsung Medical Center, Sungkyunkwan University School of Medicine;; 5 Department of Urology, Kangbuk Samsung Hospital, Seoul, Republic of Korea.

**Keywords:** Upper urinary tract cancer, Locally-advanced, Adjuvant chemotherapy, Competing risk analysis, Propensity score-matching.

## Abstract

**Objective**: To determine whether adjuvant chemotherapy (ACH) influences cancer-specific mortality, bladder cancer-specific mortality, and other-cause mortality in patients with locally advanced upper tract urothelial carcinoma (UTUC) following radical nephroureterectomy (RNU) through the use of competing risk analysis.

**Methods**: Among 785 patients with UTUC who underwent RNU from 1994 through 2015, we analyzed 338 individuals with locally advanced UTUC (pathologic T3-T4 and/or positive lymph nodes) without distant metastases. Patients were classified into two groups according to receipt of ACH. We performed a 1:1 propensity score-matching analysis between the ACH and no ACH group. The study endpoints were UTUC- and other cause-specific survivals. The association of potential risk factors with outcome was tested with the Fine and Gray regression model.

**Results**: During a median follow-up duration of 31.5 months, rates of UTUC- and other cause-mortalities were 32.9% (n = 79) and 8.7% (n = 21), respectively. Of note, there were no significant differences in overall survival between the observation and ACH groups according to the competing risks of death (UTUC and other causes of death). Multivariate analysis showed that only older age at surgery (≥ 65 years; hazard ratio [HR] = 1.73), multifocality (HR = 1.74), and tumor size (HR = 1.92) remained as poor predictors of UTUC-specific survival. Additionally, positive surgical margin was only identified as independent predictor of other causes of death (HR = 4.23).

**Conclusion**: In summary, postoperative chemotherapy failed to improve UTUC- and other cause-specific survival rates, based on competing risk analysis after propensity score-matching.

## Introduction

Upper urinary tract urothelial carcinoma (UTUC) is a rare and aggressive disease associated with high morbidity and mortality 1. Radical nephroureterectomy (RNU) is the mainstay of treatment for non-metastatic UTUC 2. However, the prognosis of patients with advanced stage UTUC, such as ≥ pathologic T3 (pT3) or node positive [pN (+)] disease, has not changed over the past decades 3. Patients with UTUC are diagnosed with locally advanced, high-grade disease following surgery should be considered for administration of adjuvant chemotherapy (ACH) 4. Although European Association of Urology (EAU) guidelines state that cisplatin-based ACH can exert a beneficial effect on both overall survival (OS) and disease-free survival, the current practice is mainly dependent on data from bladder cancer 2, 5-7. Thus, a definitive recommendation is unlikely until high-quality evidence from randomized prospective trials is obtained in patients with UTUC. Moreover, retrospective studies have shown conflicting results regarding the effects of ACH on oncological outcomes in patients with UTUC 8-11. In this regard, the role of ACH for patients with advanced stage UTUC remains an open question.

Because patients with UTUC are at high risk of intravesical recurrence, as well as renal insufficiency, following surgery and subsequent ACH, traditional methods of survival estimation, such as Kaplan Meier analysis or Cox proportional hazards modeling, may be inappropriate to evaluate the competing nature of these multiple causes of mortality. Competing risk analysis is a novel method of survival analysis that aims to correctly predict the marginal probability of a specific event in the presence of competing causes, providing more accurate information regarding the multiple competing events 12. However, to the best of our knowledge, there have been no reports on the role of ACH in patients with advanced stage UTUC that have utilized competing risk analysis. In the present study, we investigate the influence of ACH on survival outcomes in patients with advanced UTUC following RNU, particularly based on competing risk analysis after propensity score-matching for the first time.

## Patients and Methods

### Study population

We retrospectively reviewed clinical data from 785 patients with UTUC who underwent RNU between September 1994 and December 2015 at Samsung Medical Center. After excluding 447 patients with localized disease, we finally analyzed 338 patients with locally advanced UTUC [pathologic T3-T4 (pT3-4) and/or positive lymph nodes (pN (+))] without distant metastases (n=265 [pT3-4], n=73 [pN (+)]). We evaluated the following clinicopathological parameters: age at surgery, sex, body mass index (BMI), preoperative use of ureteroscopy, type of operation (open or laparoscopic surgery), tumor laterality, tumor size, tumor location, pathologic T and N stage, tumor grade, presence of lymphovascular invasion (LVI), surgical margin status, multifocality, bladder cuffing type, and receipt of adjuvant systemic chemotherapy (ACH).

The Institutional Review Board (IRB) at our hospital approved the current study (SMC 2018-04-011-001). Because this study was retrospectively performed, the IRB waived the requirement to document informed consent from the included patients. All procedures performed in the present study were in accordance with the Declaration of Helsinki guidelines.

### Study design

RNU with bladder cuff excision was performed according to the surgical protocol described previously 13. The type of surgery, open or laparoscopic RNU, was determined at the attending surgeons' discretion. Bladder cuff excision was typically conducted via the extravesical technique with a modified Gibson incision, by removing the entire ureter including the ureteral orifice. In cases of evidence of clinically significant lymph node enlargement on preoperative images and/or direct examination during surgery, we performed retroperitoneal lymph node dissection. Surgical tissue specimens were placed into 10% formalin solution for fixation and were then processed into paraffin blocks. Paraffin-embedded tissues were sectioned into 4-6 µm slices and mounted onto glass slides, followed by hematoxylin and eosin staining. Experts on genitourinary tract pathology carefully reviewed these slides and reported the key pathological findings. Pathologic T stage and tumor grade were determined by the 2009 American Joint Committee on Cancer staging system and the 1973 World Health Organization/International Society of Urologic Pathology consensus classification, respectively 14, 15. LVI was defined as the spread of cancer cells to the blood vessels and/or lymphatics within the upper urinary tract. Positive resection margin was defined as the presence of cancer cells at the end of the distal resection site of surgical specimens.

Patients received three to six courses of ACH based on a gemcitabine and cisplatin regimen at one month after surgery. Patients were classified into two groups according to receipt of ACH after surgery. To reduce the selection bias between patients receiving ACH or not, we performed a 1: 1 propensity score-matching analysis. Propensity scores were estimated by using a logistic regression model of the 8 covariates (sex, age at surgery, BMI, preoperative URS, LVI, tumor location, tumor size, pathologic N stage). The primary endpoint was UTUC-specific survival, and secondary endpoints were bladder cancer- and other cause-specific survivals. The patients underwent routine follow-up every three months during the first two years after surgery. Patients received follow-up every six months during the third year after surgery, and then checked annually thereafter. We typically performed history-taking, physical examination, routine laboratory tests including urine cytology, cystoscopy, chest radiography, and computed tomographic (CT) urography during the follow-up periods.

### Statistical analysis

We described the results of descriptive analyses of continuous variables as the median values with interquartile ranges (IQRs), as well as the actual numbers and proportions (%) of events. We performed the Mann-Whitney U test and chi-square test to identify the statistical differences in continuous and categorical variables, respectively. The association of potential risk factors with competing risk outcome was tested using the Fine and Gray regression model. Tumor size was non-normally distributed and was therefore transformed using log transformation prior to its inclusion in statistical analyses. We presented the results of statistical analysis as hazard ratios (HR) with 95% confidence intervals (CI). Statistically significant outcomes were indicated by a two-sided p-value less than 0.05. All statistical analysis in this study was executed by the Statistics and Data Center at Samsung Medical Center using SAS version 9.4 (SAS Institute Inc., Cary, NC, USA).

## Results

**Figure 1:**
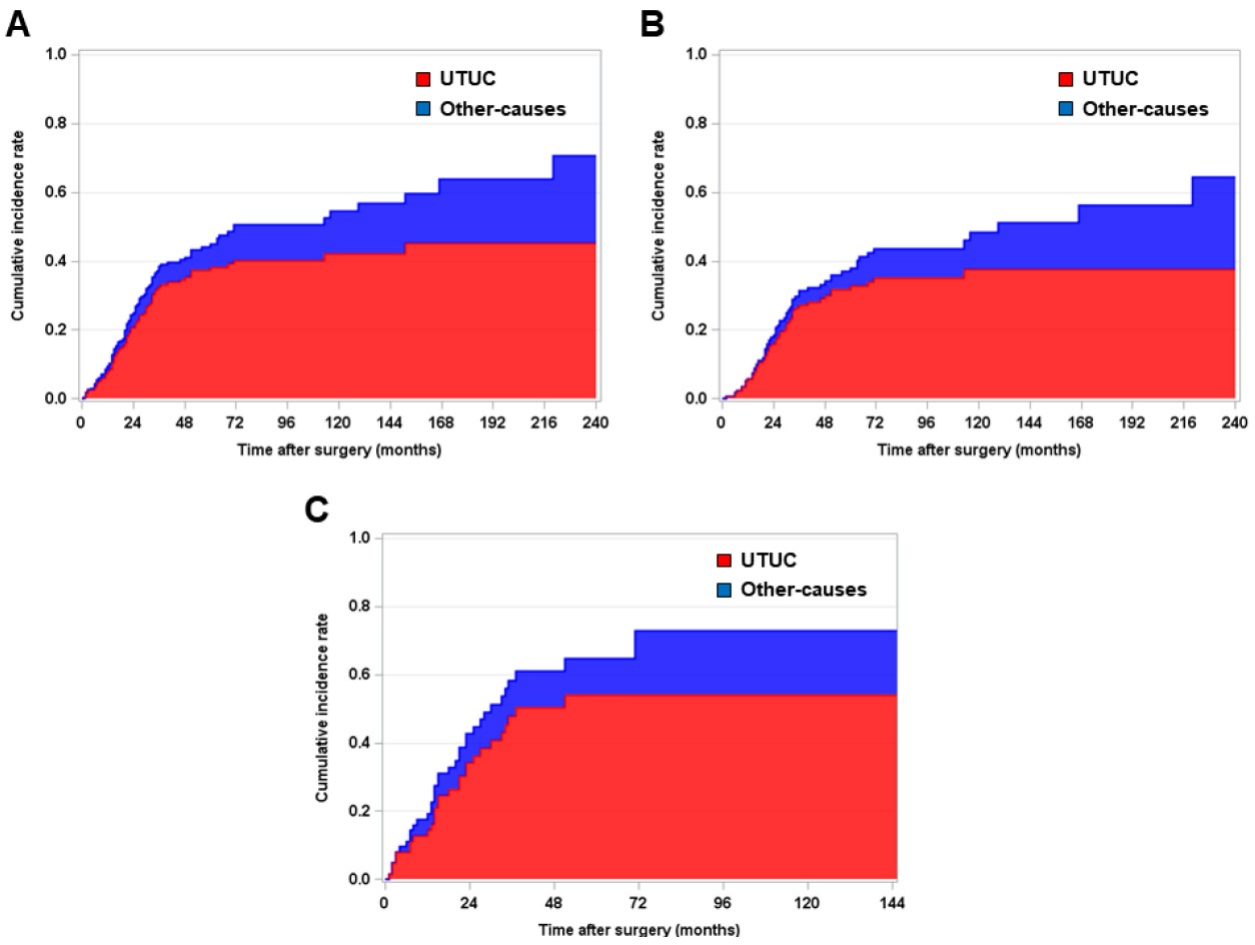
Kaplan-Meier survival curve analysis for estimating competing risk of death in patients with **(A)** pT3-4 and/or pN (+) and **(B)** pT3-4 and pN (-) and **(C)** pT any N (+) upper urinary tract urothelial carcinoma who underwent radical nephroureterectomy.

Among the entire group of 338 patients, 42.6% (n = 144) received ACH following RNU. To minimize the selection bias between the ACH and no ACH group, we performed a 1: 1 propensity score-matching analysis. The baseline characteristics of the pre-propensity (n = 338) and post-propensity (n = 240) populations are shown in Table 1. After propensity score-matching analysis, no variables were significantly different between the ACH group (n = 120) and no ACH group (n = 120). During a median follow-up period of 31.5 months (IQR = 16.0-65.0), UTUC-specific death and other-causes of death rates were 32.9% (n = 79) and 8.7% (n = 21), respectively. Fig. 1 shows graphic illustrations of competing risk analysis according to the cause of death, such as UTUC- and other-causes specific, in the overall, pT3-4 and pN (-), and pT any and pN (+) populations.

**Figure 2:**
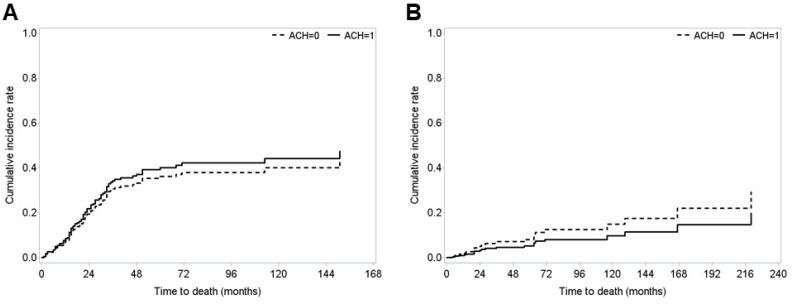
Cumulative incidence rates of **(A)** UTUC-specific death and **(B)** other-causes of death in patients with pT3-4 and/or pN (+) upper urinary tract urothelial carcinoma (UTUC) who underwent radical nephroureterectomy (RNU) according to receipt of adjuvant chemotherapy (ACH), using competing risk analysis. All survival analysis was performed after a 1:1 propensity score-matching between the ACH group and no ACH group.

Notably, we found no significant differences in survival outcomes between the observation and ACH groups in patients with pT3-4 and/or pN (+) according to the competing risks of death, UTUC and other-causes of death, respectively (Fig. 2). We also found no differences in survival outcomes between patients in the observation and ACH groups in patients with pT3-4 and pN (-) stage according to the competing risks of UTUC-specific and other-causes of death (Fig. S1). Finally, there were no statistical differences between patients in the observation and ACH groups among those with stage pN (+) disease, according to the competing risks of death, UTUC-specific and other-causes of death (Fig. S2).

We performed multivariate analysis using the Fine and Gray competing risks regression model to identify the predictors of survival outcomes with respect to the cause of death. In patients with stage pT3-4 and/or pN (+) UTUC, older age at surgery (≥ 65 years; HR = 1.73, 95% CI = 1.07-2.78), tumor size (HR = 1.92, 95% CI = 1.17-3.14) and multifocality (HR = 1.74, 95% CI = 1.04-2.92) remained as poor predictors of UTUC-specific survival (Table 2). Positive resection margin (HR = 4.23, 95% CI = 1.32-13.53) was only identified as a predictive factor of other causes of death. Conversely, no statistical significance was observed for ACH as an independent prognosticator. In patients with stage pT3-4 and pN (-) UTUC, only older age at surgery (≥ 65 years; HR = 1.87, 95% CI = 1.08-3.23) was proved as poor predictive factor for UTUC-specific survival, but no variable was identified as a predictor of other-causes of death. Similar to the results for the overall population, the use of ACH was not identified as a predictor for any competing risk of death (Table 3). In patients with stage pN (+) UTUC, we could not perform multivariate competing risk analysis owing to the small sample size (n = 44) (data not shown).

## Discussion

Extrapolating from the data on bladder cancer and limited UTUC studies, platinum-based ACH is expected to be beneficial in patients with advanced stage UTUC 16. However, there are currently insufficient data regarding the oncological role of ACH in patients with advanced UTUC, without level I evidence, as well as inconsistent results of retrospective studies. Necchi *et al*. 10 recently analyzed 1,544 patients with UTUC from a multicenter cohort and reported that no difference was observed in all-cause mortality between patients receiving ACH and those receiving observation following surgery. Kim *et al*. 8 also found no significant disease-specific or overall survival benefits associated with ACH in 138 patients who underwent RNU for locally advanced UTUC (pT3/4 or pN (+)). Conversely, Seisen and colleague evaluated 3,253 individuals with pT3/4 and/or pN (+) who received ACH or observation following RNU, and found that ACH was significantly associated with OS benefits in both the overall population (HR = 0.77) and all subgroups evaluated 11. A research group in Japan performed propensity score-matched analysis in high-risk UTUC patients, and showed that there was no statistical difference in 5-year cancer-specific survival (CSS) between patients with ACH and those without ACH (69.0% in the RNU and ACH group vs. 58.9% in the no ACH group [P = 0.030]) 9. More recently, a multicenter, randomized study (the POUT trial) has been performed to prove the benefit of ACH following surgery in patients with locally advanced UTUC. Although there were significant improvements in disease-free survival (HR 0.49 [95% CI=0.31 - 0.76]) and recurrence-free survival (HR 0.49 [95% CI=0.30 - 0.78]) in ACH group, the results are still immature to adopt as a convincing evidence of beneficial role of ACH in patients with UTUC. Therefore, the role of ACH has remained poorly defined for the management of patients with high-risk UTUC following surgery.

In the present study, we performed a competing risk analysis and first reported that there were no significant differences in UTUC-specific, bladder cancer-specific, and other causes of mortality between patients with advanced UTUC who received ACH and those who did not receive ACH after definitive surgery. Moreover, a multivariate competing risks regression model revealed that ACH was not an independent prognosticator in UTUC-specific, bladder cancer-specific, and other-cause death. Subgroup analysis also showed no significant differences between the observation and ACH groups, particularly among patients with stages pT3-4 and pN (-) and pT any and pN (+), according to competing risk of death. Conceptually, competing risk indicates an alternative outcome if a patient has the opportunity to experience one of several mutually exclusive events, and the occurrence of one event can prohibit the experience of any other event 17. Because the conventional methods for survival analyses are not designed to reflecting the competing nature of different causes of mortality, these methods are not appropriate to accurately estimate the individual risk of the event of interest, such as mortality from unrelated causes 18. For instance, patients with UTUC are at a risk of death from intravesical recurrence (or bladder cancer). Moreover, chemotherapy-related toxicity may compromise the survival outcomes in patients with renal impairment or older age, increasing other-cause mortality. In this regard, prognosis should be analyzed by considering this potential risk of bias. Therefore, our data based on a competing risk analysis, which is of more significant clinical importance, confirmed that postoperative chemotherapy could not improve the outcomes of the UTUC-, bladder cancer- and other cause-associated survival in patients with advanced UTUC.

Despite the lack of studies in patients with high-risk UTUC, Gandaglia and colleagues first presented the results of competing risk analysis for predicting disease-specific mortality, bladder cancer-related mortality, and other-cause mortalities according to age and stage in patients with localized UTUC who underwent RNU 19. The authors reported that 18.1% (n = 1797), 31.2% (n = 3090), and 9.1% (n = 891) and 3090 (31.2%) individuals died of UTUC-specific, bladder cancer-specific, and other-cause mortalities, respectively 19. These authors also found that age, tumor stage, female sex, type of surgery, grade, and tumor location were significantly associated with worse cancer-specific survival, and observed that ureteral tumor, stage, and tumor grade were related to bladder cancer-specific death 19. Inman *et al*. 20 also reported that 46% of patients died owing to the competing risks by analyzing 168 patients with UTUC, and showed that preoperative predictors of cancer-specific mortality and competing mortality were invasive tumor characteristics (HR = 3.97; P < 0.001) and older age (HR = 1.07; P < 0.001). However, there are currently no studies based on competing risk analysis in patients with advanced stage UTUC. These studies can provide a better graphical tool for risk stratification of patients with UTUC undergoing RNU by estimating UTUC, bladder cancer and other-cause mortality, respectively, in the context of competing risk analysis. From a clinical standpoint, this novel approach to survival estimation can be useful for both clinicians and patients with UTUC in terms of initial counseling, decision-making, and surveillance planning after RNU.

We acknowledge that the present study is not devoid of limitations. First, as a retrospective data, this study has unavoidable drawbacks, such as selection bias especially who was suitable for cisplatin-based chemotherapy, and inconsistent data collection during the study period. Second, although comorbid disease, such as diabetes and chronic kidney disease can influence the long-term survival outcomes in patients who received systemic chemotherapy, there was a lack of detailed information regarding comorbid diseases in the population of the present study. Third, this study was performed by using single center data, and therefore; multicenter study should be conducted to validate our results and to provide more solid conclusions regarding the role of ACH in patients with locally advanced UTUC. Despite these disadvantages, our data highlight the ineffective impact of ACH on survival outcomes, especially based on competing risk analysis, in patients with advanced UTUC following RNU.

In summary, through competing risk analysis following propensity-score matching, we demonstrated that postoperative chemotherapy did not improve UTUC-specific and other causes-specific survival, in patients with locally advanced UTUC who underwent RNU. These results can offer practical information for clinicians regarding treatment decision making in these patients, who are at high risk of death due to competing causes.

## Supplementary Material

Supplementary figures.

## Figures and Tables

**Table 1:** Baseline demographics of patients with advanced upper urinary tract carcinoma who underwent radical nephroureterectomy and adjuvant chemotherapy: Pre and post propensity score matching data.

Variables	*Pre-propensity score match*				*Post-propensity score match*			
	Total	No ACH	ACH	P-value	Total	No ACH	ACH	P-value
No. of patients	338 (100.0)	193 (57.1)	145 (42.9)		240 (100.0)	120 (50.0)	120 (50.0)	
Age at systemic therapy								
Median (IQR)	65 (57-72)	68 (58-74)	62 (56-68)	< 0.001	63 (56 - 70)	62 (55 - 72)	64 (58 - 69)	0.435
< 65 years	161 (47.6)	73 (37.8)	88 (60.7)	< 0.001	129 (53.7)	64 (53.3)	65 (54.2)	0.873
≥ 65 years	177 (52.4)	120 (62.2)	57 (39.3)		111 (46.3)	56 (46.7)	55 (45.8)	
Sex								
Male	245 (72.7)	136 (70.5)	109 (75.2)	0.323	174 (72.5)	87 (72.5)	87 (72.5)	1.000
Female	93 (27.3)	57 (29.5)	36 (24.8)		66 (27.5)	33 (27.5)	33 (27.5)	
Body mass index (kg/m^2^)								
Median (IQR)	24.1 (22.3-25.9)	23.8 (22.0 - 25.5)	24.3 (22.8-26.3)	0.064	24.2 (22.7 - 26.1)	24.1 (22.6 - 26.1)	24.3 (22.9 - 26.1)	0.732
< 25	220 (65.1)	133 (68.9)	87 (60.0)	0.107	146 (60.8)	74 (61.7)	72 (60.0)	0.768
≥ 25	118 (34.9)	60 (31.1)	58 (40.0)		94 (39.2)	46 (38.3)	48 (40.0)	
Preoperative URS								
No	194 (57.4)	119 (61.7)	75 (51.7)	0.094	137 (57.1)	70 (58.3)	67 (55.8)	0.674
Yes	144 (42.6)	74 (38.3)	70 (48.3)		103 (42.9)	50 (41.7)	53 (44.2)	
Operation type								
Open	202 (59.8)	117 (60.6)	85 (58.6)	0.736	138 (57.5)	71 (59.2)	67 (55.8)	0.600
Laparoscopy	136 (40.2)	76 (39.4)	60 (41.4)		102 (42.5)	49 (40.8)	53 (44.2)	
Tumor laterality								
Right	161 (47.6)	99 (51.3)	62 (42.8)	0.152	111 (46.2)	59 (49.2)	52 (43.3)	0.392
Left	177 (52.4)	94 (48.7)	83 (57.2)		129 (53.8)	61 (50.8)	68 (56.7)	
Tumor size (cm)	4.0 (2.7-5.8)	4.0 (2.7-5.8)	4.0 (2.8-5.2)	0.751	4.0 (2.5 - 5.5)	4.0 (2.5 - 5.7)	4.0 (2.5 - 5.1)	0.872
Tumor location								
Renal pelvis	187 (55.3)	116 (60.1)	70 (48.3)		128 (53.3)	66 (55.0)	62 (51.7)	
Ureter	103 (30.5)	51 (26.4)	53 (36.5)	0.091	77 (32.1)	36 (30.0)	41 (34.2)	0.787
Both	48 (14.2)	26 (13.5)	22 (15.2)		35 (14.6)	18 (15.0)	17 (14.1)	
Tumor grade								
G1-2	108 (32.0)	65 (33.7)	43 (29.7)	0.479	78 (32.5)	41 (34.2)	37 (30.8)	0.532
G3	230 (68.0)	128 (66.3)	102 (70.3)		162 (67.5)	79 (65.8)	83 (69.2)	
LVI								
Absence	225 (66.6)	134 (69.4)	90 (62.1)	0.200	153 (63.7)	78 (65.0)	75 (62.5)	0.647
Presence	113 (33.4)	59 (30.6)	55 (37.9)		87 (36.3)	42 (35.0)	45 (37.5)	
Resection margin								
Negative	318 (94.1)	182 (94.3)	136 (93.8)	1.000	224 (93.3)	111 (92.5)	113 (94.2)	0.593
Positive	20 (5.9)	11 (5.7)	9 (6.2)		16 (6.7)	9 (7.5)	7 (5.8)	
Multifocality								
No	244 (72.2)	141 (73.1)	103 (71.1)	0.713	177 (73.7)	89 (74.2)	88 (73.3)	0.878
Yes	94 (27.8)	52 (26.9)	42 (28.9)		63 (26.3)	31 (25.8)	32 (26.7)	
Pathologic N stage								
pN (-)	265 (78.4)	155 (80.3)	110 (75.9)	0.350	178 (74.2)	89 (74.2)	89 (74.2)	1.000
pN (+)	73 (21.6)	38 (19.7)	35 (24.1)		62 (25.8)	31 (25.8)	31 (25.8)	
Cause of death								
UTUC	110 (32.6)	62 (32.1)	48 (33.3)		79 (32.9)	36 (30.0)	43 (35.8)	
Other causes	31 (9.2)	21 (10.9)	10 (7.0)		21 (8.7)	13 (10.8)	8 (6.6)	

ACH, adjuvant chemotherapy; IQR, interquartile ratio; URS, ureterorenoscopy; LVI, lymphovascular invasion; UTUC, upper tract urothelial carcinoma.

**Table 2:** Multivariate analyses of predictors of cancer-specific, bladder cancer-specific and other cause-specific survival in patients with pT3-4 and/or pN (+) urothelial carcinoma of upper urinary tract.

Variables	UTUC			Other causes		
	HR	95% CI	*P*	HR	95% CI	*P*
Adjuvant chemotherapy						
No	Reference			Reference		
Yes	1.14	0.72 - 1.80	0.559	0.64	0.27 - 1.49	0.298
Age at surgery						
< 65 years	Reference			Reference		
≥ 65 years	1.73	1.07 - 2.78	0.023	1.35	0.54 - 3.40	0.515
Tumor size	1.92	1.17 - 3.14	0.009	0.59	0.24 - 1.50	0.274
Tumor grade						
G1 - 2	Reference			Reference		
G3	1.27	0.75 - 2.14	0.373	1.46	0.48 - 4.40	0.505
LVI						
Absence	Reference			Reference		
Presence	1.51	0.89 - 2.53	0.119	1.18	0.54 - 2.55	0.672
Margin						
Negative	Reference			Reference		
Positive	0.51	0.16 - 1.61	0.250	4.23	1.32 - 13.53	0.015
Multifocality						
None	Reference			Reference		
Yes	1.74	1.04 - 2.92	0.035	1.11	0.41 - 2.98	0.833
Pathologic N stage						
pN (-)	Reference			Reference		
pN (+)	1.34	0.79 - 2.29	0.277	1.81	0.66 - 4.96	0.246

UTUC, upper tract urothelial carcinoma; HR, hazard ratio; CI, confidence interval; URS, ureterorenoscopy; LVI, lymphovascular invasion.

**Table 3:** Multivariate analyses of predictors of cancer-specific, bladder cancer-specific and other cause-specific survivals in patients with pT3-4 and pN (-) urothelial carcinoma of upper urinary tract.

Variables	UTUC			Other causes		
	HR	95% CI	P	HR	95% CI	P
**Adjuvant chemotherapy**						
No	Reference			Reference		
Yes	1.34	0.74 - 2.41	0.325	0.46	0.14 - 1.44	0.184
**Age at surgery**						
< 65 years	Reference			Reference		
≥ 65 years	1.87	1.08 - 3.23	0.025	0.91	0.28 - 2.96	0.875
**Tumor grade**						
G1 - 2	Reference			Reference		
G3	1.39	0.76 - 2.54	0.279	1.28	0.34 - 4.78	0.708
**LVI**						
Absence	Reference			Reference		
Presence	1.77	0.97 - 3.21	0.061	0.67	0.22 - 2.06	0.488
**Multifocality**						
None	Reference			Reference		
Yes	1.79	0.91 - 3.55	0.091	2.29	0.74 0 7.04	0.148

UTUC, upper tract urothelial carcinoma; HR, hazard ratio; CI, confidence interval; LVI, lymphovascular invasion.
