# Supplementary material for: Role of Adjuvant Chemotherapy in Advanced Stage Upper Urinary Tract Urothelial Carcinoma after Radical Nephroureterectomy: Competing Risk Analysis after Propensity Score-Matching
Source: J Cancer. 2019 Nov 17;10(27):6896–902. doi: 10.7150/jca.34103 (PMC6909941; doi:10.7150/jca.34103)

Supporting information

Fig S1. Cumulative incidence rate of (A) UTUC-specific death and (B) other-causes of death in patients with pT3–4 and pN (–) upper urinary tract urothelial carcinoma (UTUC) who underwent radical nephroureterectomy (RNU) according to receipt of adjuvant chemotherapy (ACH), using competing risk analysis. All survival analysis was performed after a 1:1 propensity score-matching between the ACH group and no ACH group.

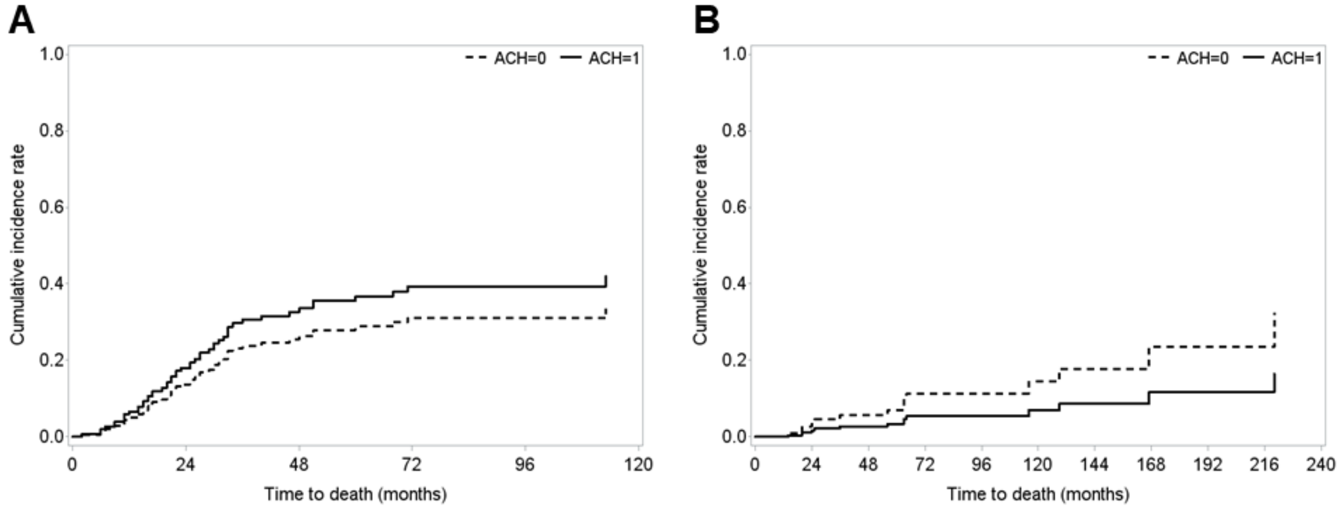

Fig S2. Cumulative incidence rate of (A) UTUC-specific death and (B) other-causes of death in patients with pT any N (+) upper urinary tract urothelial carcinoma (UTUC) who underwent radical nephroureterectomy (RNU) according to receipt of adjuvant chemotherapy (ACH), using competing risk analysis. All survival analysis was performed after a 1:1 propensity score-matching between the ACH group and no ACH group.

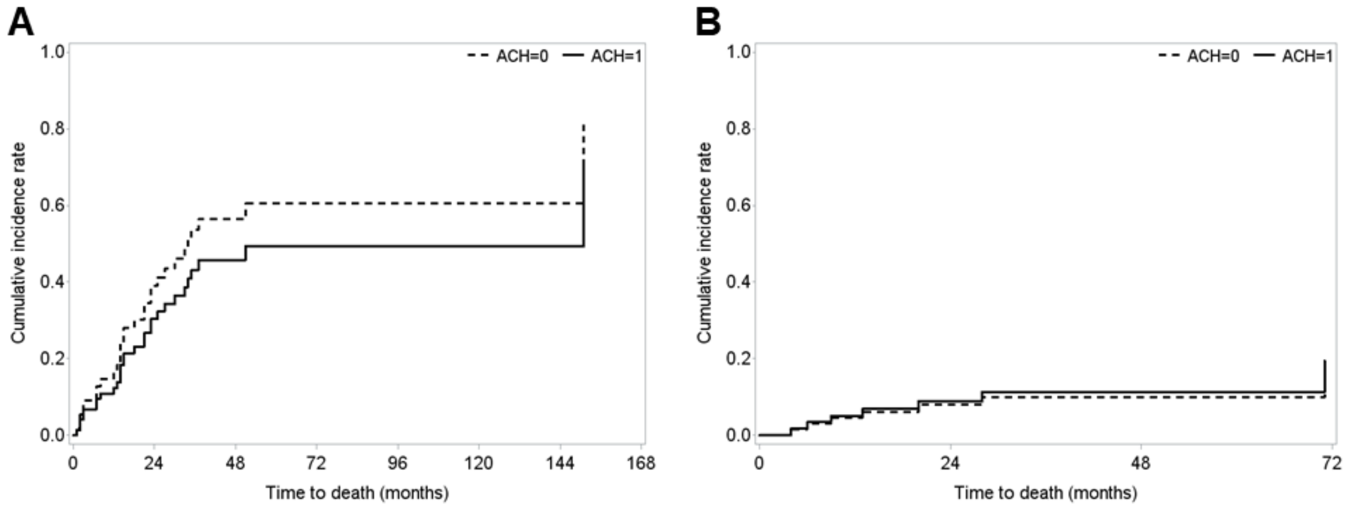

Supplement: Supplementary file 1 — Supplementary figures. [file jcav10p6896s1.pdf]
